# Experimental observations of rapid Maize streak virus evolution reveal a strand-specific nucleotide substitution bias

**DOI:** 10.1186/1743-422X-5-104

**Published:** 2008-09-24

**Authors:** Eric van der Walt, Darren P Martin, Arvind Varsani, Jane E Polston, Edward P Rybicki

**Affiliations:** 1 Department of Molecular and Cell Biology, University of Cape Town, Cape Town, South Africa; 2 Institute of Infectious Disease and Molecular Medicine, University of Cape Town, Cape Town, South Africa; 3 Electron Microscope Unit, University of Cape Town, Cape Town, South Africa; 4 University of Florida, Interdisciplinary Centre for Biotechnology Research, Bradenton, USA

## Abstract

**Background:**

Recent reports have indicated that single-stranded DNA (ssDNA) viruses in the taxonomic families *Geminiviridae*, *Parvoviridae *and *Anellovirus *may be evolving at rates of ~10^-4 ^substitutions per site per year (subs/site/year). These evolution rates are similar to those of RNA viruses and are surprisingly high given that ssDNA virus replication involves host DNA polymerases with fidelities approximately 10 000 times greater than those of error-prone viral RNA polymerases. Although high ssDNA virus evolution rates were first suggested in evolution experiments involving the geminivirus maize streak virus (MSV), the evolution rate of this virus has never been accurately measured. Also, questions regarding both the mechanistic basis and adaptive value of high geminivirus mutation rates remain unanswered.

**Results:**

We determined the short-term evolution rate of MSV using full genome analysis of virus populations initiated from cloned genomes. Three wild type viruses and three defective artificial chimaeric viruses were maintained *in planta *for up to five years and displayed evolution rates of between 7.4 × 10^-4 ^and 7.9 × 10^-4 ^subs/site/year.

**Conclusion:**

These MSV evolution rates are within the ranges observed for other ssDNA viruses and RNA viruses. Although no obvious evidence of positive selection was detected, the uneven distribution of mutations within the defective virus genomes suggests that some of the changes may have been adaptive. We also observed inter-strand nucleotide substitution imbalances that are consistent with a recent proposal that high mutation rates in geminiviruses (and possibly ssDNA viruses in general) may be due to mutagenic processes acting specifically on ssDNA molecules.

## Background

Most research on virus evolution has focussed on RNA viruses, which are generally subject to relatively high rates of mutation due to their dependence on error-prone DNA dependent RNA polymerases. Accordingly, RNA viruses have been shown to evolve at rates between 10^-3 ^to 10^-5 ^substitutions per site per year (subs/site/year) [1-4]. In contrast – and consistent with the hypothesis that polymerase fidelity influences evolution rates – double stranded DNA (dsDNA) bacteriophages, papillomaviruses and polyomaviruses evolve at rates in the region of 10^-9 ^subs/site/year [5,6]. Intriguingly, and possibly contradicting the premise that polymerase fidelity is the major universal determinant of evolution rates, figures closer to those of RNA viruses (~10^-4 ^subs/site/year) have been reported for the small single stranded DNA (ssDNA) anelloviruses [7-9] and parvoviruses [10-12]. Furthermore, direct estimates of the basal or biochemical rates at which mutations occur during each replication cycle of ssDNA bacteriophages have also indicated that these rates approach those of RNA viruses [5,13] For a good general review on the topic of virus mutation and evolution rates see [14].

The ssDNA geminiviruses represent extremely important threats to commercial agriculture and basic subsistence farming throughout the tropical and temperate regions of the world [15-18]. The geminiviruses are a highly diverse group comprising more characterised species than any other virus family [19]. Although interest in geminivirus evolution has, until recently, been largely focussed on the undeniably important role of recombination in the generation of novel species and strains [20-25], it is the accumulation of point mutations that is the ultimate source of diversity within the family.

Very little is known about the timescales over which geminivirus diversification has occurred. The apparent absence of any members of the most divergent geminivirus genus – the mastreviruses – in the New World strongly suggests that the earliest geminiviruses only evolved after the break-up of Gondwanaland ~100 million years ago [26]. Additionally, all available phylogenetic evidence indicates that the geminiviruses currently found in the Americas were introduced there much more recently: most extant New World geminiviruses probably evolved from one or a few progenitor begomoviruses that were possibly introduced as recently as 20 000 years ago along with human colonists from Asia via the Bering land bridge [27], and a few species originating in the middle East and Asia have been accidentally released in the Americas in modern times [28,29].

Importantly, indirect estimates of geminivirus evolution rates and direct experimental measurement of geminivirus mutation frequencies both indicate that, as is the case for some other ssDNA virus groups, geminiviruses are evolving at an unexpectedly rapid rate. Duffy & Holmes [30], using Bayesian coalescent based analysis of geminiviruses causing Tomato yellow leaf curl disease (eight separate old world begomovirus species), reported that the average genome-wide rate at which mutations have been fixed in the genomes of these viruses over the past 20 years has been approximately 2.88 × 10^-4 ^subs/site/year. While the credibility interval of this estimate is quite broad, it is 95% certain that the last common ancestor of the eight species studied existed within the past 41 000 years. It is noteworthy that the most probable date for the origin of these viruses, which represent approximately the same breadth of diversity as that currently observable amongst new-world begomoviruses, is between 3000 and 9000 years ago – a figure that fits well with the hypothesis that humans and begomoviruses may have colonised the Americas at approximately the same time.

Although only two direct experimental measurements of geminivirus mutation frequencies appear in the literature, both confirm that these viruses are capable of evolving at rates of between 10^-3 ^and 10^-4 ^subs/site/year. The first, using a "biologically cloned" MSV population maintained for up to four years in both maize and in a *Coix *sp., estimated a genome-wide evolution rate of between 2.6 × 10^-4 ^and 5.5 × 10^-4 ^subs/site/year [31] within individual infected plants. The second, using infectious cloned tomato yellow leaf curl China virus (TYLCCV) isolates maintained for between 60 and 120 days in *Nicotiana benthamiana *and tomato plants, detected evolution rates of between 1.4 × 10^-3 ^and 2.2 × 10^-3 ^subs/site/year in a genome region that included the *rep *gene and the intergenic region [32].

Two reports of high-frequency reversions of specific non-lethal deleterious mutations in the *rep *genes of MSV [33,34] and isolates of various begomovirus species [35] indicate that the basal rate at which mutations occur in geminivirus genomes may be orders of magnitude higher than the rate at which mutations become fixed within these genomes. At a particular genomic site analysed in one of these experiments, a highly adaptive reversion mutation was detectable in 5/8 independent MSV infections within 10 days of inoculation [33] implying that the virus is capable of adaptive evolution rates rivalling those of even the most rapidly evolving RNA viruses.

Thus, the population wide evolution rates estimated for geminiviruses by Duffy and Holmes [30] are slightly lower than evolution rates directly observed within individual infections [31,32], which are in turn lower than mutation rates implied by mutation frequency studies involving highly adaptive reversion mutations [33-35]. These differences in estimated evolution rates probably reflect the effects of population size and selection pressure on the rate at which mutations become fixed in a population [13]. Selection operates more effectively on larger populations, with advantageous mutations rising to fixation and deleterious mutations being purged quicker than for small populations [36]. Furthermore, it has been experimentally verified in various systems that, consistent with the popular theoretical concept of scaling a fitness peak, rates of evolutionary adaptation to new environments are initially rapid but eventually slow down and level off [37-42]. This is because as a sequence ascends a fitness peak the fraction of possible advantageous mutations permitting upward movement becomes progressively smaller. The fraction reaches zero as the peak is attained, at which point the evolution rate should match the rate of selectively neutral genetic drift. As a result of these factors, short-term evolution rates estimated from small populations of a virus species, such as those measured within individual infected plants over a few years, will be somewhere between the basal rate at which mutations occur for that species and the long-term rate at which the species is evolving over tens or hundreds of years [13].

To accurately measure the rate at which MSV genomes accumulate mutations over periods of a few years, and to study the relationship between fitness and evolution rate, we studied nucleotide substitutions arising in defective mutant and wild-type MSV genomes during infections of maize and sugarcane. Three of the genomes analysed were unusual in that they were low-fitness laboratory constructed MSV chimaeric viruses comprising genome components we knew to be specifically maladapted to survival in maize [23,43]. In addition to estimating the short-term MSV evolution rate within individual hosts, we present evidence that MSV exhibits strand specific nucleotide substitution imbalances that are consistent with a recent proposal by Duffy and Holmes [30] that high mutation rates in ssDNA viruses are due to mutagenic processes that specifically affect ssDNA molecules.

## Results and discussion

### Mutations occur at high frequencies during MSV infections

**Figure 1:**
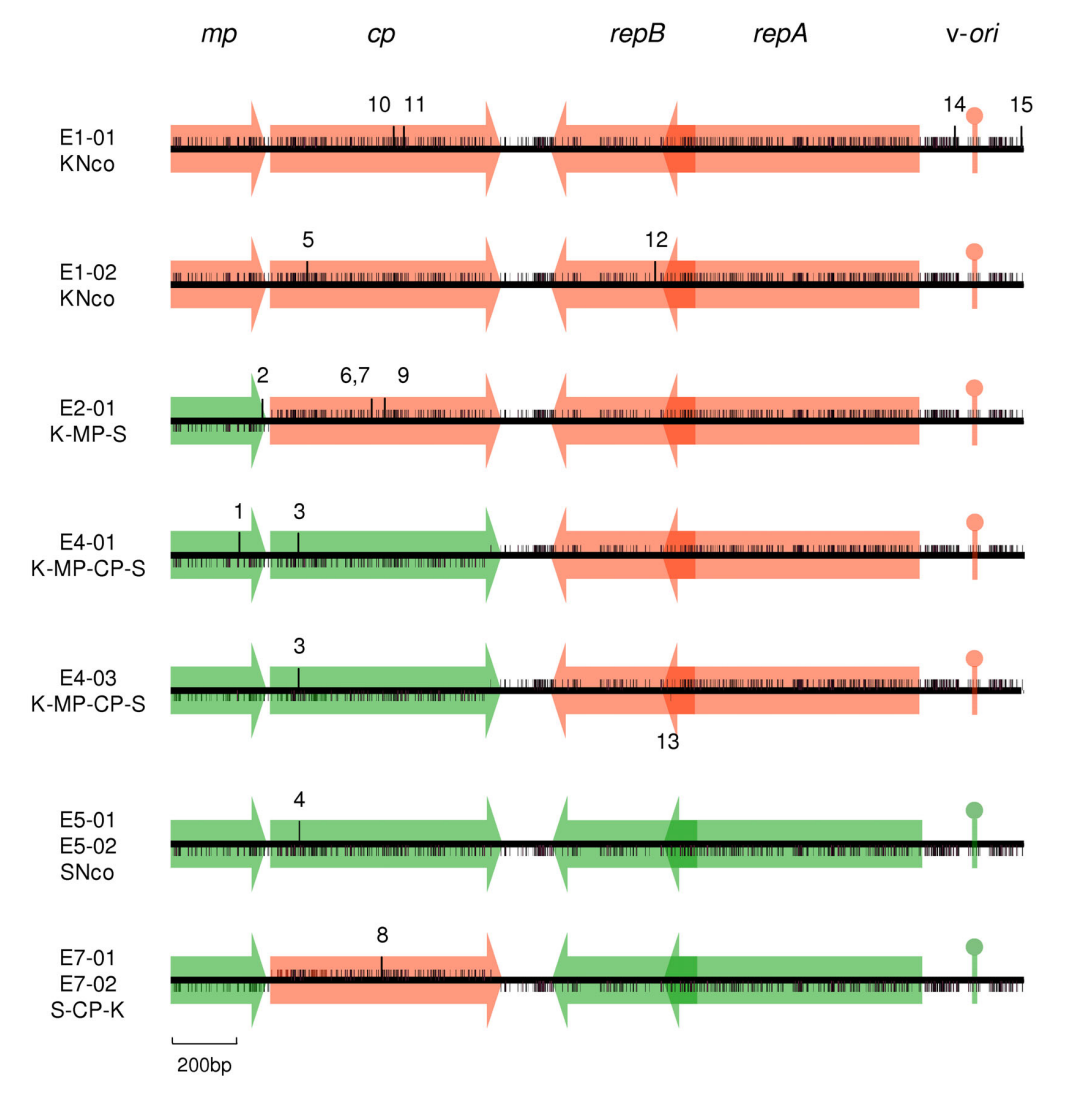
**Mutations in MSV-Kom/-Set parental and chimaeric viruses.** Short vertical lines above or below the centre line indicate homology at informative sites to either MSV-Kom or MSV-Set, respectively. Long vertical lines above the centre line represent positions not homologous to either MSV-Kom or MSV-Set sequence (i.e. mutated sites). Mutations are numbered, and refer to those listed in Additional file 1. The positions of ORFs and the virion-strand replication origin stem-loop sequence are indicated in shaded red (MSV-Kom) or green (MSV-Set). The diagrams are to scale.

With the intention of studying evolution rates and patterns of nucleotide substitution in MSV, sweetcorn plants were initially agroinoculated with clones of three wild-type MSV strains – MSV-Tas, MSV-Kom and MSV-Set – and three defective laboratory constructed recombinant viruses – K-MP-S, K-MP-CP-S and S-CP-K (Figure 1). All are described in detail by van der Walt *et al*. [43].

We used two approaches to avoid the severe population bottlenecks that were likely to occur during insect transmission in the course of our experiments. Our first approach, used with all viruses other than MSV-Tas, utilised three plants infected with each virus to initiate serial transmissions via leafhopper, with each transmission lasting several days and involving tens of leafhoppers. Our second approach, used with MSV-Tas, was to avoid serial leafhopper transmissions altogether. To achieve this, a single sugarcane plant (cultivar Uba) was infected with the wild-type isolate MSV-Tas via leafhopper transmission from an agroinoculated sweetcorn plant [44], and maintained in an infected state for five years. Although MSV-Tas was originally isolated from wheat, it produces relatively severe symptoms in sugarcane [44], indicating that it was not particularly maladapted to this perennial host.

Following twelve passages through sweetcorn over a one-year period, no obvious changes in symptomatology were observed for any of the serially transmitted viruses (data not shown). At the end of the one-year period, viral genomes were cloned from one symptomatic plant infected with each of the viruses. Full-length genomic sequences were obtained for two individual MSV genomic clones from each plant, except for K-MP-S, for which only one genome was sequenced. Similarly, seventeen full-length MSV-Tas genomes were cloned and sequenced from the five year old infection of sugarcane.

**Figure 2:**
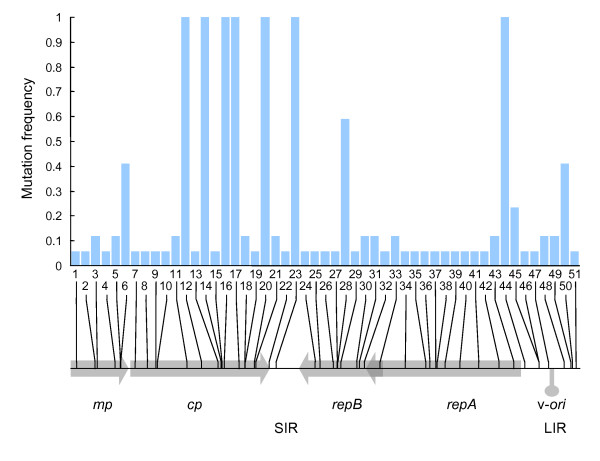
**Mutation frequencies in seventeen MSV-Tas derived genomes isolated after five years of maintenance in sugarcane.** The histogram represents the proportions of the 17 analysed genomes that carried the different mutations. Beneath the histogram, the positions of ORFs and the virion-strand replication origin are indicated in shaded grey. The genomic locations of the 51 analysed mutations are indicated by vertical black lines overlaying the genome map. Mutation numbers correspond to those in Additional file 2. *mp *= movement protein gene; *cp *= coat protein gene; *RepA*+*RepB *= replication associated protein gene;*repA *= RepA gene.

Figure 1 and 2 respectively show the positions of all of the mutations identified in the nine genome sequences from maize and the 17 genome sequences from sugarcane, while Additional files 1 and 2 respectively detail the nucleotide and protein sequence context and the specific sequence changes in each individual clone from maize and sugarcane. All of the genomes sequenced contained at least one mutation with respect to the original parental viruses; the most mutations in any single genome was four (E1-01, MSV-Kom; E2-01, K-MP-S) for the maize viruses and 18 (SC-E02) for the sugarcane viruses. Besides three identical clone pairs (E5-01 and E5-02; E7-01 and E7-02; E3 and F7) all 20 remaining genomes were unique.

A total of 66 different mutations were detected overall: 15 in the viruses from maize and 51 in the viruses from sugarcane. Two of these were deletion mutations (mutation 12 in E1-02 and mutation 33 in SC-E-02 and F10; Figures 1 and 2 respectively) and one was an insertion mutation (mutation 44 found in all clones from sugarcane). Whereas the insertion mutation was at a site in the LIR that seems to tolerate insertions and deletions in related MSV isolates, both the deletion mutations are likely to be lethal in that they cause *rep *frame shifts that should result in the expression of seriously truncated and partially mistranslated Rep proteins. For example, a 16 nt deletion in SC-E-02 and F10 would be predicted to result in loss of the *rep *intron acceptor site and premature termination of *repA *some thirty codons before the normal stop site. It is very unlikely that SC-E-02 and F10 could somehow express a functional Rep despite this deletion in that both also carry a substitution mutation (mutation 30 in Figure 2 and Additional file 2) that introduced a premature stop codon at Rep position 257.

While these deletion mutations should disable the viruses carrying them, many of the 63 nucleotide substitution mutations are probably neutral in that the vast majority did not alter any nucleotide or amino acid sequence motifs with either known or suspected functionality and, based on their having PAM250 scores > 1 [45], most of the predicted amino acid changes are probably relatively conservative. Notable exceptions were three independent mutations that disrupted the most distal of three potential C-sense TATA boxes in clones E1-01 (mutation 14 in Figure 1 and see Additional file 1), SC-E02, SC-F01, C5, F10 and F5 (mutations 45 and 46 in Figure 2 and see Additional file 2).

### MSV displays evolution rates similar to those of other ssDNA viruses

Whereas the average evolution rate of the nine genome sequences from maize was 7.4 × 10^-4 ^subs/site/year (20 substitutions in 24183 nucleotides sequenced), the average rate for the seventeen sequences from sugarcane was 7.9 × 10^-4 ^subs/site/year (180 substitutions in 45713 nucleotides sequenced). While these rates are approximately half those recently determined for the related begomovirus, TYLCCV. (Ge *et al.*, 2007), they are between 3- and 4-fold higher than a previous estimate of MSV evolution rates [31].

It is not entirely surprising that our evolution rate estimate is higher than that made by Isnard *et al*. [31] because whereas our estimates are based on mutational distances from known progenitor sequences, theirs are based on distances from a population consensus sequence. Had we used a consensus of the 17 MSV-Tas derived clones instead of the MSV-Tas progenitor sequence itself, our evolution rate estimate for the viruses maintained in sugarcane would have been 2.6 × 10^-4 ^subs/site/year – only 1.1-fold higher than the lower rate estimated by Isnard *et al*. [31].

It is important to note that the MSV evolution rates we have measured should be considered "short-term small-population" evolution rate estimates, and they are almost certainly an over-estimation of longer-term population-wide rates [13]. Whereas an ideal evolution rate estimate would be the rate at which mutations become fixed within the global MSV population, our short-term small-population estimates more closely reflect the rate at which mutations accumulate in MSV genomes during a single infection. This rate provides an indication of the maximum rate at which MSV could evolve; however, it is the slower rate at which such mutations become fixed, through drift and positive selection, that determines how rapidly large MSV populations evolve over tens or hundreds of years.

Nevertheless, based on the evolution rate estimates reported here and elsewhere [30-32], it is becoming increasingly apparent that geminiviruses are probably evolving as fast as some RNA viruses[3,4,46,47] and orders of magnitude faster than dsDNA viruses [48,49]. This represents a significant departure from the natural assumption that the synthesis of geminivirus genomes by host DNA polymerases [50,51] implies relatively error-free virus replication and therefore mutation rates similar to those experienced by plant genomic DNA [52,53]. At least two other diverse ssDNA viruses seem to have nucleotide substitution rates in the range of 10^-4 ^subs/site/year – parvoviruses [11,12] and anelloviruses [7] – which implies that high mutation rates may be a common, if not universal, feature among ssDNA viruses.

### Nucleotide substitution biases suggest a possible cause of high MSV mutation rates

Because of our relatively scant understanding of plant DNA replication in general, and more specifically of the host factors involved in geminivirus replication [51,54], the mechanisms underlying the surprisingly high mutation rates seen in geminiviruses remain a topic of speculation. There are, however, some clues about where to start looking. As early as 1997, Roossinck [53] noted that since replicating geminivirus DNA is apparently not methylated [55] it is possible that normal host mechanisms for mismatch repair may not operate during their replication [56]. Both Ge *et al*. [32] and Duffy and Holmes [30] made the same proposal. Duffy and Holmes [30] suggested two additional possibilities: i) because geminivirus DNA is only transiently double-stranded during rolling-circle replication, it may not be suitable for base-excision repair; ii) the biased substitution patterns may be explained either by spontaneous deamination – potentially more likely to occur in ssDNA [57-59] – or by the action of deaminating host enzymes [60].

One way to explore these alternative possibilities is to examine substitution biases. Duffy and Holmes [30] detected high rates of C→T and G→A transitions that were possibly indicative of increased C and G deamination rates. As deamination rates are probably higher for ssDNA, this was taken to imply that high begomovirus mutation rates might be at least partially attributable to the considerable fraction of their life-cycles spent in ssDNA form.

However, another way of using substitution biases as an indicator of ssDNA specific mutagenic processes is to compare the substitution rates of complementary substitutions. If ssDNA is specifically prone to a mutagenic process that, for example, results in an increased rate of T→C transitions, then there should be evidence of significantly more T→C transitions on the virion strand (the only strand that spends any appreciable time in a single stranded state) than on the complementary strand. As the two strands are complementary, one need only compare rates of complementary T→C and A→G transitions on the virion strand to determine whether the mutagenic mechanism in question is more active on ssDNA.

We examined the 63 substitution mutations to determine whether there was any evidence of substitution biases in MSV. Table 1 lists the number of observed mutations of each substitution type, as well as the expected frequencies taking initial genome-wide nucleotide frequencies into account. We found that G→T transversions were over-represented in both the maize and sugarcane evolution experiments, and that this over-representation was highly significant when either the MSV-Tas sequence dataset was analysed alone (chi square p < 10^-8^) or when all the mutation data from both experiments were considered collectively (chi square p = 5.4 × 10^-7^; Table 1). Though not statistically significant in our relatively small dataset, the complementary C→A changes appeared to be consistently under-represented. That there is such an obvious imbalance in the complementary G→T and C→A transversions strongly supports the hypothesis that a mutagenic process causing G→T transversions on the virion DNA strand (the strand predominantly found in single stranded form) is at least partially responsible for higher than expected mutation rates in MSV.

**Table 1:** Analysis of nucleotide substitution and mutation distribution biases in MSV genome sequences derived from evolution experiments in maize (MSV-Kom, -Set and defective recombinant sequences) and sugarcane (MSV-Tas sequences).

	MSV-Kom,-Set and defective recombinants			MSV-Tas			All mutants analysed		
	
Substitution (V-sense)	Obs.	Exp.	Χ^2 ^p-value	Obs.	Exp.	Χ^2 ^p-value	Obs.	Exp.	Χ^2 ^p-value
**Transversions**									
A → C	2	1.18	0.43	3	4.26	0.54	5	5.39	0.87
A → T	1	1.18	0.86	3	4.26	0.54	4	5.39	0.55
C → A	0	1.08	0.28	2	3.65	0.39	2	4.62	0.22
C → G	1	1.08	0.94	5	3.65	0.48	6	4.62	0.52
G → C	1	1.22	0.84	4	3.87	0.95	5	4.90	0.96
G → T	3	1.22	0.09	13	3.87	**< 10**^-8^	16	4.90	**5.4 × 10**^-7^
T → A	0	1.19	0.25	3	4.26	0.54	3	5.39	0.30
T → G	0	1.19	0.25	4	4.26	0.90	4	5.39	0.55
**Transitions**									
A → G	3	1.18	0.08	4	4.26	0.90	7	5.39	0.48
C → T	1	1.08	0.94	3	3.65	073	4	4.62	0.77
G → A	2	1.22	0.46	3	3.87	0.66	5	4.90	0.96
T → C	0	1.19	0.25	2	4.26	0.27	2	5.39	0.14
Ins	0			1			1		
Del	1			1			2		
**GC Content**									
A/T→G/C	5	4.7	0.50	13	17	0.07	18	21.6	**0.05**
G/C→A/T	6	4.6	0.50	21	15	0.07	27	19.0	**0.05**
**Transitions (Ts) vs transversions (Tv)**									
Tv	8	9.3	0.45	37	32.7	0.19	45	42	0.42
Ts	6	4.7	0.45	12	16.3	0.19	18	21	0.42
Ts/Tv	0.75	0.5		0.32	0.5		0.40	0.5	
**Coding vs non-coding genome regions**									
Coding	13	12.4	0.67	42	42.1	0.99	55	54.4	0.85
Non-coding	2	2.6	0.67	9	9.9	0.99	11	11.6	0.85
**Synonymous (S) vs non-synonymous (N) mutations**									
N	6	10.1	**6 × 10**^-3^	27	30.4	0.18	33	40.5	**0.012**
S	7	2.9	**6 × 10**^-3^	12	8.6	0.18	19	11.5	**0.012**
dN/dS	0.24	1.0		0.64	1.0		0.49	1.0	

Probably as a consequence of the high rate of G→T mutations, there was evidence of a significant trend towards lower GC content over the course of the evolution experiments when all mutations were collectively considered (chi square p = 0.05). However, despite the high G→T mutation bias, there was no significant trend in favour of transversion mutations over transition mutations (Table 1).

Whereas guanine and cytosine deamination of virion sense ssDNA has been cited as a possible cause of the increased frequencies of G→A and C→T transitions observed in begomoviruses [30], the over representation of G→T transversions we have observed in MSV is probably caused by some other form of damage to single stranded MSV DNA. One possible mechanism is the oxidation of guanine into 8-oxoguanine which then base-pairs with adenine during replication and causes G→T transversions. Formation of 8-oxoguanine is known to be the most common cause of spontaneous G→T transversions in many organisms [61-64]. That an increased rate of G→T transversions has been associated with time spent as ssDNA [65-67] fits very well with the notion that increased rates of MSV mutation may be at least partially attributable to either increased rates of 8-oxoguanine formation or decreased rates of 8-oxoguanine lesion repair in virion sense ssDNA.

### Negative selection predominates but some mutations may be adaptive

Mutations were distributed among coding and non-coding sites more or less as expected, given their relative numbers (Table 1). The ratio of non-synonymous to synonymous substitutions (dN/dS) was significantly less than one when either the maize experiment dataset (collectively including sequences derived from wt MSV-Kom, MSV-Set and the defective chimaeric viruses) was considered in isolation (chi square p = 6.0 × 10^-3^) or when all data was collectively considered (chi square p = 1.2 × 10^-2^; Table 1). This indicated that the sequences, particularly those from maize, were most likely evolving under a predominance of negative (or purifying) rather than positive (or diversifying) selection. Unfortunately our datasets contained insufficient diversity and too few sequences for the kinds of site-by-site selection analyses that enable detection of individual sites evolving under positive selection against a background of negative selection [68,69].

We nevertheless thought it probable that evidence of adaptive evolution might be detectable amongst the mutations found in the defective chimaeric virus dataset. Disruptions of specific interactions between CP and MP and between CP and some other as yet unidentified viral genome region(s) are apparently responsible for the reduced fitness of these chimaeric viruses [23,43]. We hypothesised that fitness losses caused by transferring *mp*, *cp *or *mp-cp *coding regions between MSV-Kom and MSV-Set might have been partially recouped through compensatory mutations within the *mp-cp *cassette that restored damaged interactions either within the *mp-cp *cassette, or between the cassette and the remainder of the MSV genome. It was anticipated that the most obvious sign of such "repaired interactions" would be mutations within the *mp-cp *cassettes of defective chimaeric viruses that changed identity from that of one parental sequence to the other.

However, only one mutation (13 in Figure 1 and see Additional file 1) out of eight detected in the defective chimaeric viruses represented a change from one wild-type parental sequence to the other. This mutation was one of four (mutations 6, 7 and 9 in Figure 1 were the others) that occurred at sites that were polymorphic between MSV-Kom and MSV-Set. This is close to the expected number (4/3 = 1.3) of conversions between MSV-Kom and MSV-Set polymorphisms if one assumes random mutation. In the context of reports that some MSV mutants either revert or experience compensatory mutations at high rates to restore fitness [33-35] and that MSV can adaptively overcome host resistance within a period of about a year [31], we were surprised by this result. Together with the fact that we observed no changes in the symptomatology of any of our defective chimaeric viruses after a year in maize, this lends support to the results of our dN/dS analyses (Table 1) indicating that few, if any, of the observed genetic changes were beneficial evolutionary adaptations.

The only indication of positive selection that we found in the defective chimaeric virus dataset was a significantly elevated number of substitutions in the *mp-cp *cassette of these viruses. We compared the distribution of mutations between the *mp-cp *and *repA-repB *coding regions in the defective MSV-Kom/-Set chimaeras with the mutation distributions seen in the progeny genomes of wild type MSV-Kom, -Set, and -Tas infections. In both the MSV-Kom/Set and the MSV-Tas datasets, neither the *mp-cp *cassette nor the *repA-repB *cassette contained disproportionately more mutations than could be accounted for by chance. Similarly, the number of mutations in the *repA-repB *cassette of the defective chimaeric viruses was not significantly higher than expected by chance. However, the *mp-cp *cassette of these viruses contained eleven times more substitutions per site than did the rest of their genomes (chi square p-value = 0.014). On the other hand, considering that only two of these substitutions resulted in (relatively conservative) non-synonymous changes (mutations 2 and 7, see Additional file 1) any positive selection that may have occurred was likely to have been acting on noncoding aspects of the DNA sequences such as those identified by Shepherd *et al*. [33].

## Conclusion

We have presented evidence from controlled evolution experiments lasting up to five years that indicates that MSV experiences high rates of evolution close to those recently approximated in shorter term experiments for another geminivirus species [32]. Collectively these results add credibility to reports that on a long term global scale geminiviruses may be evolving at rates as high as those reported for many RNA viruses [30]. For the first time we show strand-specific substitution biases which directly indicate that at least some of the mutational processes underlying high MSV evolution rates are acting preferentially on ssDNA. While the increased mutability of ssDNA may neatly account for disparities between the evolution rates of ssDNA and dsDNA viruses, proof of this may ultimately require a detailed comparative analysis of the individual impacts of all mutagenic reactions and repair pathways acting on single and double stranded DNA molecules.

## Methods

### Virus isolates, plasmids, bacterial strains, plants and leafhoppers

Agroinfectious clones of MSV-Kom, MSV-Set, K-MP-S, K-MP-CP-S and S-CP-K [43,70] have been described previously. *Agrobacterium tumefaciens *C58C1 [pMP90] was used to deliver viral DNA to maize cv. Jubilee (sweetcorn) seedlings by agroinoculation as described by Martin *et al*. [71]. The MSV-Tas infected sugarcane plant (cultivar Uba) used in this study was the same as that mentioned in a previous publication [44]. A virus-free *Cicadulina mbila *colony maintained at the University of Cape Town since 1990 was used as a source of leafhoppers during transmissions [72].

### Leafhopper transmission of viruses

*C. mbila *leafhoppers and infected plants were maintained isolated in purpose-built cages (410 mm × 410 mm × 710 mm, w × d × h) at approximately 21°C with indirect natural light augmented by Grolux™ fluorescent tubes for 12 hours per day. Each cage contained plants infected with a single virus genotype. Initially three 25-day-old plants infected by agroinoculation with each of MSV-Kom, MSV-Set, K-MP-S, K-MP-CP-S, and S-CP-K were placed in separate isolation cages with c.a. 100 adult leafhoppers and three uninfected 8-day-old maize seedlings per cage. When symptoms became visible on new plants the older plants were removed from the cage and replaced with seedlings; this cycle was repeated approximately monthly. The entire experiment lasted for 12 months, during which the viruses were passaged through 12 generations of maize plants.

Initiation of a MSV-Tas infection in a single sugarcane plant (cv. Uba) by leafhopper transmission from an agroinoculated maize plant is described in [44]. This infected sugarcane plant was maintained for five years at 25°C with 16 hours of light per day provided by Grolux fluorescent tubes.

### Isolation, cloning and sequencing of viral DNA

Replicative form, double-stranded virus DNA was extracted from plants as described by Palmer *et al. *[73]. Isolated virus genomes were ligated either into the *Bam*HI site of pUC18 using standard techniques (all clones labelled Ex-0y and SC-Ex-0x) [74] or using phi29 DNA polymerase (TempliPhi™, GE Healthcare, USA) as described previously [75,76] (all clones labelled Cx, Ex and Fx where C, E, F indicate that clones were obtained from different shoots). Briefly, the amplified concatamers were digested with *Bam*HI, to yield ~2.7-kb linearised viral genomes which were ligated with linearised pGEMZf+ (Promega Biotech). Individual genome sequences were determined by the University of Cape Town DNA Sequencing Service (Molecular and Cell Biology Department, UCT), the University of Florida Interdisciplinary Center for Biotechnology Research DNA sequencing service, or commercially sequenced (Macrogen Inc., Korea) using the primer set described by Owor *et al*. [75]. All mutations were verified by at least two sequencing runs. All parental virus clones were re-sequenced in both directions.

### Sequence analysis

The expected frequency for a given substitution of nt. X for nt. Y (f^E^_X→Y_) was calculated assuming all substitution types were equally likely, as f ^E^_X→Y _= (P_X _× M)/3 where P_X _is the fractional proportion of nucleotide X (= A, G, T or C) in the parental sequence, and M is the total number of observed mutations. Significant deviation from the expected number of mutations of a given type was tested using a 2 × 2 chi square test (ie. observed and expected substitutions numbers of a particular type × observed and expected substitution numbers of all other types pooled). Expected transition (Ts) and transversion (Tv) frequencies were calculated by summing the expected frequencies of the relevant substitutions. Significant deviation of observed Tv and Ts values from those expected under the null hypothesis of Tv/Ts = 2 (i.e. all mutations occur at the same frequency irrespective of whether they are transitions or transversions) was calculated using a 2 × 2 chi square test.

To calculate the proportions of nonsynonymous mutations per nonsynonymous site (dN) and proportions of synonymous mutations per synonymous site (dS), the numbers of nonsynonymous and synonymous sites in each coding region were obtained using the Datamonkey web-server [61]. The numbers of synonymous and nonsynonymous mutations in each coding region were determined manually. Deviation of observed dN and dS values from those expected assuming a dN/dS ratio of 1 (i.e. neutrality) was tested using a 2 × 2 chi square test.

## List of abbreviations used

CP: Coat protein; cp: Coat protein gene; dsDNA: double stranded DNA; LIR: Long intergenic region; MP: movement protein; mp: movement protein gene; MSV: Maize streak virus; NSP: Nuclear shuttle protein; ORF: Open reading frame; PCR: Polymerase chain reaction; Rep: replication associated protein; *rep *: replication associate protein gene; SD: Standard deviation; SIR: Short intergenic region; ssDNA: Single stranded DNA; TYLCV: Tomato yellow leaf curl virus.

## Competing interests

The authors declare that they have no competing interests.

## Authors' contributions

EvdW conceived the study, carried out the experiments, analysed the data and prepared the manuscript. AV helped carry out the experiments. DPM helped analyse the data and prepare the manuscript. JP helped carry out the experiments. EPR supervised the study, secured funding for its execution and helped prepare the manuscript. All authors read and approved the final manuscript.

**Table 2:** Distribution of mutations by genomic region.

Genomic region	Mutations in region	(region size)	Mutations in rest of genome	(region size)	Fold difference in sub/site		Χ^2 ^p-value
MSV-Kom/Set – parental viruses							
*mp *+ *cp*	4	(1039)	3	(1658)	2.1		0.534 †
*repA *+ *repB*	1	(1159)	6	(1538)	0.2		0.250 †
MSV-Kom/Set – chimaeras							
*mp *+ *cp*	7	(1039)	1	(1658)	11.2		0.014 † *
*repA *+ *repB*	1	(1159)	7	(1538)	0.2		0.164 †
**MSV-Tas**							
*mp *+ *cp*	22	(1039)	29	(1644)	1.20		0.617 †
*repA *+ *repB*	20	(1159)	31	(1524)	0.85		0.671 †

† Yates' chi-squared p-values; * p-value < 0.05.

## Supplementary Material

Additional file 1 Mutations in MSV-Kom, MSV-Set and defective recombinants passaged in maize. Click here for file

Additional file 2 Mutations in MSV-Tas passaged in sugarcane. Click here for file
