# Supplementary material for: Experimental observations of rapid Maize streak virus evolution reveal a strand-specific nucleotide substitution bias
Source: Virol J. 2008 Sep 24;5:104. doi: 10.1186/1743-422X-5-104 (PMC2572610; doi:10.1186/1743-422X-5-104)
Supplement: Additional file 1 — Mutations in MSV-Kom, MSV-Set and defective recombinants passaged in maize. [file 1743-422X-5-104-S1.doc]

**Additional file** 1. Mutations in MSV-Kom, MSV-Set and defective recombinants passaged in maize.

| a | Nt ^b^ | **Parent**  Clone(s) | Genomic Region  (Protein) | Sequence changes | PAM250 ^c^ |
| --- | --- | --- | --- | --- | --- |
| 1 | 221 | **K-MP-CP-S**  E4-01 | *mp* (MP) | F G S E **A** V D R R  TTTGGATCTGAAG**C**TGTGGATAGGAGG  ↓  TTTGGATCTGAAG**T**TGTGGATAGGAGG  F G S E **A** V D R R | 2 |
| 2 | 292 | **K-MP-S**  E2-01 | *mp* (MP) | G P F V **P** G Q G *  GGACCGTTCGTT**C**CAGGTCAGGGATAA  ↓  GGACCGTTCGTT**G**CAGGTCAGGGATAA  G P F V **A** G Q G * | 1 |
| 3 | 407 | **K-MP-CP-S**  E4-01  E4-07 | *cp* (CP)  (DNA-binding region) | Q A K K **P** G G K V  CAGGCGAAGAAGC**C**TGGGGGTAAGGTT  ↓  CAGGCGAAGAAGC**A**TGGGGGTAAGGTT  Q A K K **P** G G K V | 6 |
| 4 | 412 | **MSV-Set**  E5-01  E5-02 | *cp* (CP)  (DNA-binding region) | A K K P **G** G K V E  GCGAAGAAGCCTGG**G**GGTAAGGTTGAG  ↓  GCGAAGAAGCCTGG**A**GGTAAGGTTGAG  A K K P **G** G K V E | 5 |
| 5 | 436 | **MSV-Kom**  E1-02 | *cp* (CP)  (DNA-binding region) | D R P S **L** Q I Q T  GATAGGCCATCCCT**A**CAAATCCAGACA  ↓  GATAGGCCATCCCT**C**CAAATCCAGACA  D R P S **L** Q I Q T | 6 |
| 6, 7 | 640, 641 | **K-MP-S**  E2-01 | *cp* (CP)  (DNA-binding region) | T G T G **V** **M** W L V Y  ACCGGAACCGGTGT**AA**TGTGGCTGGTGTAT  ↓↓  ACCGGAACCGGTGT**TG**TGTGGCTGGTGTAT  T G T G **V** **V** W L V Y | 4, 2 |
| 8 | 672 | **S-CP-K**  E7-01  E7-02 | *cp* (CP) | T T P G **G** Q A P T  ACCACTCCCGGCG**G**ACAAGCTCCGACC  ↓  ACCACTCCCGGCG**A**ACAAGCTCCGACC  T T P G **G** Q A P T | 5 |
| 9 | 682 | **K-MP-S**  E2-01 | *cp* (CP) | G G Q A **P** T P Q T  GGCGGACAAGCTCC**G**ACCCCGCAAACT  ↓  GGCGGACAAGCTCC**T**ACCCCGCAAACT  G G Q A **P** T P Q T | 6 |
| 10 | 710 | **MSV-Kom**  E1-01 | *cp* (CP) | F A Y P **D** T L K A  TTTGCCTACCCT**G**ACACGCTAAAAGCG  ↓  TTTGCCTACCCT**C**ACACGCTAAAAGCG  F A Y P **H** T L K A | 1 |
| 11 | 743 | **MSV-Kom**  E1-01 | *cp* (CP) | A T W K **V** S R E L  GCCACATGGAAA**G**TGAGCCGGGAGCTG  ↓  GCCACATGGAAA**T**TGAGCCGGGAGCTG  A T W K **L** S R E L | 2 |
| 12 | 1540 | **MSV-Kom**  E1-02 | *repB* (Rep)  Potential *repA* polyadenylation signal  (*myb*-like transactivation domain) | N Y W Q **N** **N V D W - - - ***  AATTACTGGCAAA**A**TAATGTTGATTGG-N^320^-TAA  ↓  AATTACTGGCAAA**.**TAATGTTGATTGG-N^36^ -TAA  N Y W Q **I** **M L I G - - *** | -8 |
| 13 | 1590 | **K-MP-CP-S**  E4-03 | *repA* (RepA)  (Potential GRAB interaction domain)    *repA-repB* (Rep)  (dNTP binding motif) | *repA*, 26 bp upstream of STOP:    A Q Q E **P** E N L L  GGCCCAACAAGAAC**C**GGAAAATCTACT  ↓  GGCCCAACAAGAAC**A**GGAAAATCTACT  A Q Q E **Q** E N L L  Rep, 57 bp downstream of splice junction:  G P T R **T** G K S T  GGCCCAACAAGAAC**C**GGAAAATCTACT  ↓  GGCCCAACAAGAAC**A**GGAAAATCTACT  G P T R **T** G K S T | 0  3 |
| 14 | 2492 | **MSV-Kom**  E1-01 | LIR  C-sense gene TATA box | CTCCATTGTCTT**A**TAGTGGTTGTA  ↓  CTCCATTGTCTT**G**TAGTGGTTGTA |  |
| 15 | 2704 | **MSV-Kom**  E1-01 | LIR  *mp* START | M D P  CAAGTGCGATTC**A**TTCATGGATCC  ↓  CAAGTGCGATTC**G**TTCATGGATCC |  |

^a^ Numbers correspond to those in Error: Reference source not found.

^b^ nucleotide positions are given relative to the conserved *Bam*HI restriction site at the V1 start codon.

^c^ PAM250 scores ≥ 1 may indicate conservative amino acid changes. For each mutation, the following information is given on successive lines, from top to bottom: parental amino acid sequence (N→C); parental nucleotide sequence (5’→3’, coding strand, or virion strand if not coding); arrow(s) indicating mutation(s); mutant nucleotide sequence; mutant amino acid sequence. Residues in red indicate changes from parental sequences; affected codons are underlined. Where functional motifs are known or suspected to form a part of the region shown, they are indicated by highlighting. Stop codons are indicated by *.
