# Supplementary material for: Experimental observations of rapid Maize streak virus evolution reveal a strand-specific nucleotide substitution bias
Source: Virol J. 2008 Sep 24;5:104. doi: 10.1186/1743-422X-5-104 (PMC2572610; doi:10.1186/1743-422X-5-104)
Supplement: Additional file 2 — Mutations in MSV-Tas passaged in sugarcane. [file 1743-422X-5-104-S2.doc]

Additional file 2: Mutations in MSV-Tas passaged in sugarcane

| a | Pos^b^ | Clone | Genomic Region | Sequence changes | PAM250^c^ |
| --- | --- | --- | --- | --- | --- |
| 1 | 30  (11) | C5 | *mp* (MP) | F L L Q **P** R V P T  TTTCTTTTACAGCC**G**CGGGTACCCACA  ↓  TTTCTTTTACAGCC**T**CGGGTACCCACA  F L L Q **P** R V P T | 6 |
| 2 | 128  (44) | F10 | *mp* (MP) | I C F Y **L** L Y L W  ATTTGCTTTTACC**T**GCTTTACCTTTGG  ↓  ATTTGCTTTTACC**G**GCTTTACCTTTGG  I C F Y **R** L Y L W | -3 |
| 3 | 139  (48) | SC-E-02  E3 | *mp* (MP)  *mp* intron  T-tracts  Branch point  (Putative transmembrane domain) | L L Y L **W** V L R D  CTGCTTTACCTT**T**GGGTGCTGAGAGAT  ↓  CTGCTTTACCTT**A**GGGTGCTGAGAGAT  L L Y L **R** V L R D | 2 |
| 4 | 233  (79) | C2 | *mp* (MP) | V D R S **N** P I P N  GTGGATAGGAGCA**A**CCCTATCCCTAAT  ↓  GTGGATAGGAGCA**C**CCCTATCCCTAAT  V D R S **T** P I P N | 0 |
| 5 | 262  (89) | F7,F8 | *mp* (MP) | Q A P P **S** Q G N P  CAGGCACCACCA**A**GTCAGGGGAATCCC  ↓  CAGGCACCACCA**G**GTCAGGGGAATCCC  Q A P P **G** Q G N P | 1 |
| 6 | 263  (89) | SC-F-01,  F5,F9,C4,  C5,C6,E5 | *mp* (MP) | Q A P P **S** Q G N P  CAGGCACCACCAA**G**TCAGGGGAATCCC  ↓  CAGGCACCACCAA**T**TCAGGGGAATCCC  Q A P P **I** Q G N P | -1 |
| 7 | 339  (9) | SC-E-02 | *cp* (CP)  (Bipartite nuclear localisation signal)  (DNA-binding region) | K R K R **G** D D A N  AAGAGGAAGCGGG**G**AGATGATGCGAAC  ↓  AAGAGGAAGCGGG**T**AGATGATGCGAAC  K R K R **V** D D A N | -1 |
| 8 | 405  (31) | C2 | *cp* (CP) | G L K K **A** G S K A  GGTCTGAAGAAGG**C**TGGAAGCAAGGCC  ↓  GGTCTGAAGAAGG**T**TGGAAGCAAGGCC  G L K K **V** G S K A | 0 |
| 9 | 449  (43) | C7 | *cp* (CP) | S L Q I **Q** T L Q H  ATCCAGACACTC**C**AGCATGCTGGGTCC  ↓  ATCCAGACACTC**G**AGCATGCTGGGTCC  S L Q I **E** T L Q H | 2 |
| 10 | 458  (49) | F7 | *cp* (CP) | L Q H A **G**  S T M I  CTCCAGCATGCT**G**GGTCCACCATGATA  ↓  CTCCAGCATGCT**T**GGTCCACCATGATA  L Q H A **W** S T M I | -7 |
| 11 | 613  (100) | SC-E-02  F10 | *cp* (CP)  (DNA-binding region) | S Q A C **K** Y S N T  TCGCAAGCCTGC**A**A**G**TATTCTAACACC  ↓  TCGCAAGCCTGC**A**ATTATTCTAACACC  S Q A C **N** Y S N T | 1 |
| 12 | 691  (126) | C2,C4,C5,C6,C7,E1, E2,SC-E-02,E3,E4, E5, SC-F-01,F5,F7, F8, F9, F10 | *cp* (CP) | P T P Q **T** I F A Y  CCGACCCCACAAAC**C**ATATTTGCATAT  ↓  CCGACCCCACAA**ACG**ATATTTGCATAT  P T P Q **T** I F A Y | 3 |
| 13 | 778  (155) | F10 | *cp* (CP) | F V V K **R** R W L F  TTCGTGGTGAAACG**G**CGATGGTTGTTC  ↓  TTCGTGGTGAAACG**C**CGATGGTTGTTC  F V V K **R** R W L F | 6 |
| 14 | 792  (159) | C2,C4,C5,C6,C7,E1, E2,SC-E-02,E3,E4, E5, SC-F-01,F5,F7, F8, F9, F10 | *cp* (CP) | R W L F **N** M E T D  CGATGGTTGTTCA**A**CATGGAGACCGAC  ↓  CGATGGTTGTTCA**G**CATGGAGACCGAC  R W L F **S** M E T D | 1 |
| 15 | 800  (163) | E3 | *cp* (CP) | F N M E **T** D G R I  TTCAACATGGAG**A**CCGACGGTCGAATT  ↓  TTCAACATGGAG**C**CCGACGGTCGAATT  F N M E **P** D G R I | 0 |
| 16 | 811  (165) | C2,C4,C5,C6,C7,E1, E2,SC-E-02,E3,E4, E5, SC-F-01,F5,F7, F8, F9, F10 | *cp* (CP) | E T D G **R** I G S D  GAGACCGACGGTCG**A**ATTGGTTCGGAC  ↓  GAGACCGACGGTCG**G**ATTGGTTCGGAC  E T D G **R** I G S D | 6 |
| 17 | 886  (191) | C2,C4,C5,C6,C7,E1, E2,SC-E-02,E3,E4, E5, SC-F-01,F5,F7, F8, F9, F10 | *cp* (CP) | F H K F **T** S G L G  TTCCACAAGTTCAC**G**AGTGGGTTGGGA  ↓  TTCCACAAGTTCAC**C**AGTGGGTTGGGA  F H K F **T** S G L G | 3 |
| 18 | 919  (202) | SC-E-02  F10 | *cp* (CP) | T Q W K **N** V T D G  ACGCAGTGGAAGAA**T**GTAACGGACGGA  ↓  ACGCAGTGGAAGAA**C**GTAACGGACGGA  T Q W K **N** V T D G | 2 |
| 19 | 920  (203) | E2 | *cp* (CP) | Q W K N **V** T D G G  CAGTGGAAGAAT**G**TAACGGACGGAGGA  ↓  CAGTGGAAGAAT**T**TAACGGACGGAGGA  Q W K N **L** T D G G | 2 |
| 20 | 970  (219) | C2,C4,C5,C6,C7,E1, E2,SC-E-02,E3,E4, E5, SC-F-01,F5,F7, F8, F9, F10 | *cp* (CP) | A L Y M **V** I A P G  GCTCTGTACATGGT**C**ATTGCCCCTGGC  ↓  GCTCTGTACATGGT**T**ATTGCCCCTGGC  A L Y M **V** I A P G | 4 |
| 21 | 977  (221) | E2,F10 | *cp* (CP) | M V I A **P** G N G L  ATGGTCATTGCC**C**CTGGCAATGGCCTT  ↓  ATGGTCATTGCC**A**CTGGCAATGGCCTT  M V I A **T** G N G L | 0 |
| 22 | 1045  (244) | SC-E-02 | *cp* (CP)  *cp* STOP  V-transcript polyadenylation signal | S V G N **Q** *  AGTGTCGGCAACCA**G**TAATGAATAAAA  ↓  AGTGTCGGCAACCA**T**TAATGAATAAAA  S V G N **H** * | 3 |
| 23 | 1087 | C2,C4,C5,C6,C7,E1, E2,SC-E-02,E3,E4, E5, SC-F-01,F5,F7, F8, F9, F10 | SIR | TCTGATGAATGCT**C**AAAGCTTACATTA  ↓  TCTGATGAATGCT**G**AAAGCTTACATTA |  |
| 24 | 1290  (330) | C7 | *rep* (Rep) | E Y F E **A** N C M I  GAGTATTTCGAGG**C**AAACTGCATGATT  ↓  GAGTATTTCGAGG**G**AAACTGCATGATT  E Y F E **G** N C M I | 1 |
| 25 | 1314  (322) | SC-F-01 | *rep* (Rep) | N E M T **P** G Q L E  AATGAAATGACTC**C**AGGGCAGCTGGAGT  ↓  AATGAAATGACTC**T**AGGGCAGCTGGAGT  N E M T **L** G Q L E | -3 |
| 26 | 1386  (298) | E5 | *rep* (Rep) | G K K K **K** V Q M K  GGCAAGAAGAAGA**A**AGTTCAGATGAAG  ↓  GGCAAGAAGAAGA**G**AGTTCAGATGAAG  G K K K **R** V Q M K | 3 |
| 27 | 1404  (292) | SC-F-01 | *rep* (Rep) | V V N P **K** Y G K K  GTTGTAAATCCAA**A**ATATGGCAAGAAG  ↓  GTTGTAAATCCAA**C**ATATGGCAAGAAG  V V N P **T** Y G K K | 6 |
| 28 | 1406  (291) | SC-F-01,F7,F8, C2,C5,C7,E1,E2,E3,E4 | *rep* (Rep) | F V V N **P** **K** Y G K  TTCGTTGTAAATCC**A**A**A**ATATGGCAAG  ↓  TTCGTTGTAAATCC**T**A**A**ATATGGCAAG  F V V N **P** **K** Y G K | 6 |
| 29 | 1427  (284) | SC-E-02 | *rep* (Rep) | L V G C **Q** K E F V  TTAGTTGGCTGTCA**G**AAAGAGTTCGTT  ↓  TTAGTTGGCTGTCA**C**AAAGAGTTCGTT  L V G C **H** K E F V | 3 |
| 30 | 1508  (257) | SC-E-02, F10 | *rep* (Rep)  (*myb*-like transactivation domain) | D W S S **Y** **N E D A**  GATTGGTCTTCTTA**C**AACGAAGACGCA  ↓  GATTGGTCTTCTTA**A**AACGAAGACGCA  D W S S ***** | -8 |
| 31 | 1522  (253) | SC-E-02, F10 | *rep* (Rep)  Potential C1 polyadenylation signal  *myb*-like transactivation domain | Q N N V **D** W S S Y  CAAAATAATGTT**G**ATTGGTCTTCTTAC  ↓  CAAAATAATGTT**C**ATTGGTCTTCTTAC  Q N N V **H** W S S Y | 1 |
| 32 | 1548  (242) | C2 | *rep* (Rep)  RepA stop codon | R S L G **L** H N Y W  AGAAGCTTAGGGC**T**TCATAATTATTGG  ↓  AGAAGCTTAGGGC**G**TCATAATTATTGG  R S L G **R** H N Y W | -3 |
| 33 | 1633-1648  (242)-  (246) | SC-E-02, F10 | *repA* (RepA)  Rep intron  Acceptor site  (Potential GRAB interaction domain) | E W M **D** K L S S Q **Q M K - - *** GAATGGATGGA**T**AAATTATCCAGCCAACAGATGAAG-N^69^-TAA  ↓↓↓↓↓↓↓↓↓↓↓↓↓↓↓↓  GAATGGATGGA................**C**AGATGAAG  E W M **D** **R *** | -8 |
| 34 | 1764  (203) | F10 | *repA* (RepA)  *rep* (Rep)  pRBR protein binding domain | L C N E **S** I K D W  CTTTGTAATGAG**T**CAATCAAAGATTGG  ↓  CTTTGTAATGAG**A**CAATCAAAGATTGG  L C N E **T** I K D W | 1 |
| 35 | 1873  (166) | SC-F-01 | *repA* (RepA)  *rep* (Rep) | P Y D W **A** T K L Q  CCCTATGACTGGGC**C**ACAAAATTGCAG  ↓  CCCTATGACTGGGC**T**ACAAAATTGCAG  P Y D W **A** T K L Q | 2 |
| 36 | 1892  (160) | E3 | *repA* (RepA)  *rep* (Rep) | M I R K **E** F P Y D  ATGATTCGTAAAG**A**GTTCCCCTATGAC  ↓  ATGATTCGTAAAG**C**GTTCCCCTATGAC  M I R K **A** F P Y D | 0 |
| 37 | 1924  (149) | SC-F-01 | *repA* (RepA)  *rep* (Rep) | S H S T **S** K L E Y  TCACACTCCACTTC**A**AAGCTAGAGTAC  ↓  TCACACTCCACTTC**T**AAGCTAGAGTAC  S H S T **S** K L E Y | 2 |
| 38 | 1931  (147) | C2 | *repA* (RepA)  *rep* (Rep) | I I S H **S** T S K L  ATTATTTCACACT**C**CACTTCAAAGCTA  ↓  ATTATTTCACACT**A**CACTTCAAAGCTA  I I S H **Y** T S K L | -3 |
| 39 | 1975  (132) | SC-E-02 | *repA* (RepA)  *rep* (Rep) | G N S E **K** K P S K  GGAAATTCTGAAAA**G**AAACCTTCAAAA  ↓  GGAAATTCTGAAAA**T**AAACCTTCAAAA  G N S E **N** K P S K | 1 |
| 40 | 2152  (73) | C2 | *repA* (RepA)  *rep* (Rep) | K P V R **I** T D S R  AAACCGGTAAGAAT**C**ACTGACTCAAGG  ↓  AAACCGGTAAGAAT**A**ACTGACTCAAGG  K P V R **I** T D S R | 5 |
| 41 | 2257  (38) | E5 | *repA* (RepA)  *rep* (Rep) | M I W E **L** V G R W  ATGATTTGGGAGCT**C**GTTGGTCGTTGG  ↓  ATGATTTGGGAGCT**A**GTTGGTCGTTGG  M I W E **L** V G R W | 6 |
| 42 | 2297  (25) | F7 | *repA* (RepA)  *rep* (Rep) | Y P H C **P** E N P E  TATCCACACTGTC**C**AGAAAATCCAGAA  ↓  TATCCACACTGTC**A**AGAAAATCCAGAA  Y P H C **Q** E N P E | 0 |
| 43 | 2337  (12) | SC-E-02, F10 | *repA* (RepA)  *rep* (Rep) | R Q F S **H** R N V N  CGTCAATTCTCA**C**ACAGGAACGTTAAC  ↓  CGTCAATTCTCA**T**ACAGGAACGTTAAC  R Q F S **Y** R N V N | 0 |
| 44 | 2435 | C2,C4,C5,C6,C7,E1, E2,SC-E-02,E3,E4, E5, SC-F-01,F5,F7, F8, F9, F10 | LIR  TATA box  Inverse repeat of partial ori stem sequence | CTATATCAACCGG**-**TTGCGCCTTCGAA  ↓  CTATATCAACCGG**C**TTGCGCCTTCGAA |  |
| 45 | 2468 | SC-E-02,  SC-F-01, C5,F10 | LIR  TATA box | CGCTCCCCCTTTT**A**TAGTGGTTGTTTA  ↓  CGCTCCCCCTTTT**T**TAGTGGTTGTTTA |  |
| 46 | 2469 | F5 | LIR  TATA box | GCTCCCCCTTTTATAGTGGTTGTTTAT  ↓  GCTCCCCCTTTTAGAGTGGTTGTTTAT |  |
| 47 | 2523 | C4 | LIR  Invariant nonanucleotide at V-*ori*  Inverted repeat sequence | AAGGCGCGCAATAATATTACCGCGCCTT  ↓  AAGGCGCGCTATAATATTACCGCGCCTT |  |
| 48 | 2601 | SC-E-02,  F10 | LIR  AT-tracts | TCGGTTCTGCTTT**G**TCTGATTTATCTA  ↓  TCGGTTCTGCTTT**T**TCTGATTTATCTA |  |
| 49 | 2637 | F7,F8 | LIR | AATCTAAAGAAACCGGTCCCCTGCGACT  ↓  AATCTAAAGAAACTGGTCCCCTGCGACT |  |
| 50 | 2646 | E1,E2,E3,E4,C7,F7, F8 | LIR  TATA box | AAACCGGTCCCCTGCGACTATAAATTGT  ↓  AAACCGGTCCCCTCCGACTATAAATTGT |  |
| 51 | 2667 | E2 | LIR  TATA box  V2 start | AAATTGTTTCACAAGTGCGATTCATTTA  ↓  AAATTGTTTCACAGGTGCGATTCATTTA |  |

^a^ Numbers correspond to those in Figure 2

^b^ Nucleotide positions are given relative to the conserved BamHI restriction site at the V1 start codon, while amino acid positions are shown in parentheses

^c^ PAM250 scores ≥ 1 may indicate conservative amino acid changes. For each mutation, the following information is given on successive lines, from top to bottom: parental amino acid sequence (N→C); parental nucleotide sequence (5’→3’, coding strand, or virion strand if not coding); arrow(s) indicating mutation(s); mutant nucleotide sequence; mutant amino acid sequence. Residues in red indicate changes from parental sequences; affected codons are underlined. Where functional motifs are known or suspected to form a part of the region shown, they are indicated by highlighting. Stop codons are indicated by *.
